# Can routine clinical data identify older patients at risk of poor healthcare outcomes on admission to hospital?

**DOI:** 10.1186/s13104-017-2705-7

**Published:** 2017-08-10

**Authors:** Kinda Ibrahim, Charlotte Owen, Harnish P. Patel, Carl May, Mark Baxter, Avan A. Sayer, Helen C. Roberts

**Affiliations:** 1 Academic Geriatric Medicine, University of Southampton, Southampton General Hospital, Tremona Road, Mailpoint 807, Southampton, SO16 6YD UK; 2 NIHR CLAHRC Wessex, Faculty of Health Science, University of Southampton, Highfield, Southampton, SO17 1BJ UK; 3 University Hospital Southampton NHS Foundation Trust, Tremona Road, Southampton, SO16 6YD UK; 4 Ageing Geriatrics & Epidemiology, Institute of Neuroscience, The Medical School, Newcastle University, Framlington Place, Newcastle upon Tyne, NE2 4HH UK; 5 NIHR Newcastle Biomedical Research Centre in Ageing and Chronic Disease, The Medical School, Newcastle University, Newcastle upon Tyne NHS Foundation Trust, Framlington Place, Newcastle upon Tyne, NE2 4HH UK

**Keywords:** Risk assessment, Hospital, Older patients, Qualitative data, Healthcare outcomes, Medical notes

## Abstract

**Objective:**

Older patients who are at risk of poor healthcare outcomes should be recognised early during hospital admission to allow appropriate interventions. It is unclear whether routinely collected data can identify high-risk patients. The aim of this study was to define current practice with regard to the identification of older patients at high risk of poor healthcare outcomes on admission to hospital.

**Results:**

Interviews/focus groups were conducted to establish the views of 22 healthcare staff across five acute medicine for older people wards in one hospital including seven nurses, four dieticians, seven doctors, and four therapists. In addition, a random sample of 60 patients’ clinical records were reviewed to characterise the older patients, identify risk assessments performed routinely on admission, and describe usual care. We found that staff relied on their clinical judgment to identify high risk patients which was influenced by a number of factors such as reasons for admission, staff familiarity with patients, patients’ general condition, visible frailty, and patients’ ability to manage at home. “Therapy assessment” and patients’ engagement with therapy were also reported to be important in recognising high-risk patients. However, staff recognised that making clinical judgments was often difficult and that it might occur several days after admission potentially delaying specific interventions. Routine risk assessments carried out on admission to identify single healthcare needs included risk of malnutrition (completed for 85% patients), falls risk (95%), moving and handling assessments (85%), and pressure ulcer risk assessments (88%). These were not used collectively to highlight patients at risk of poor healthcare outcomes. Thus, patients at risk of poor healthcare outcomes were not explicitly identified on admission using routinely collected data. There is a need for an early identification of these patients using a valid measure alongside staff clinical judgment to allow timely interventions to improve healthcare outcomes.

**Electronic supplementary material:**

The online version of this article (doi:10.1186/s13104-017-2705-7) contains supplementary material, which is available to authorized users.

## Introduction

Many older inpatients are at high risk of adverse clinical outcomes such as longer length of stay, reduced physical function, increased dependency, admission to a care home, readmission to hospital and death [1–3]. It is well recognised that patient factors including physical function, illness severity, cognition, comorbidity, presenting medical diagnosis, polypharmacy, frailty and age can influence the outcomes of hospital admission [4]. However, the organisation of care can also impact on healthcare outcomes.

Best clinical practice includes the identification of older individuals at risk of poor healthcare outcomes routinely in order to optimise patient-centred service delivery [5, 6]. Routine early identification of high-risk patients may help healthcare professionals assess individuals’ needs and provide appropriate interventions [7]. However, it is unclear whether and how those people are identified in current practice. The purpose of this study was to define current practice in one hospital with regard to the use of routine clinical data to identify patients at high-risk of poor healthcare outcomes.

## Main text

### Methods

Interviews and focus groups were used to investigate the current practice of clinical staff in identifying older patients at risk of poor healthcare outcomes. In addition, quantitative data was abstracted from patients’ clinical records to contextualise the study patients and routine admission practice. The study was conducted in five acute medical wards (120 beds) for older people in one hospital in England including three female and two male wards.

### Qualitative data obtained from staff interviews and focus groups

In-depth semi-structured interviews and focus groups were conducted by the first author (KI) with the assistance of a moderator (CO) with a range of healthcare staff on the study wards. Interview schedules were designed to obtain information on the current roles and experiences of each participant in relation to identifying high-risk patients (see Additional file 1). Written consent was obtained and the conversations were audio-recorded. Participants were recruited until no more new concepts emerged (i.e. sampling saturation) [8].

Interviews and focus groups were transcribed verbatim, password protected, and anonymised. Data was analysed thematically using Framework method [9]. A descriptive framework containing three main themes (described below) was identified from the initial analysis of the transcripts. Two researchers (KI, HCR) completed the analysis with regular discussion. A software program (NVivo 10) [10] was used to facilitate data analysis.

### Quantitative data abstraction from clinical records

A sample of 60 patients’ clinical records were reviewed by two researchers (KI & CO) to characterise the older patients, contextualise the wards and describe the usual care provided on admission. Demographic data on age, sex, date of admission, domicile status, and reasons for admission were obtained. Information recorded about diagnosis, resuscitation status and routine assessment measures applied to patients on admission were collected. Exclusion criteria included patients who were at end of life or had been admitted for less than three days at the time of data collection.

Quantitative data were analysed using descriptive statistics and the statistical software package IBM SPSS statistics 22. Data were summarised using mean (standard deviation, SD), median (interquartile range, IQR) and number (percent, %) as appropriate. The association between clinical outcomes (e.g. length of stay and discharge destination) and risk assessments scores was completed using Chi square or linear regression as appropriate.

## Results

### Qualitative results

A total of five focus groups and three interviews were conducted with 22 staff participants across the five study wards with different sex, professional bands, and years of experience. The sample comprised: seven nurses, four dieticians, four therapists (two physiotherapists and two occupational therapists) and seven doctors: two consultants, two foundation year (FY) doctors (doctors within the first 2 years post-graduation), and three specialty registrars (trainee doctors specialising in Geriatric Medicine). Three main themes developed from the analysis reflecting participants’ views and experiences of identifying older patients at risk of poor healthcare outcomes (Table 1).

**Table 1:** Themes, sub-themes and illustrative data extracts

Themes	Sub-themes	Quotations from participants
Clinical judgment	Reasons for admission	Quote 1. The risk of deterioration is based on the reason for admission, so you can make a judgement on their acute situation and the likelihood of further deterioration. (Consultant 2)
	Familiarity with patient	Quote 2. Very often I know the patients, because they’ve previously been under my care. So from that picture, it’s relatively easy to understand where the patient is within their life course trajectory of becoming increasingly frail with old age. (Consultant 1)
	Patient’s general condition	Quote 3. I think you can. Just the general, the patient’s general condition is an indicator. (Nurse 2)
	Visible frailty	Quote 4. We try and say clinical judgement as well. So if you, because sometimes the patient won’t necessarily fall exactly into a MUST category, especially if they haven’t been weighed. I speak to nurses on the ward, do try and promote you know clinical judgement. (Dietician 4)
		Quote 5. I don’t do the referral, but I’ll ask for one if they’re visibly just very cachectic, or they look very malnourished and look very frail as well as not eating much. (Registrar 1)
	Managing at home	Quote 6. When they’d say oh they’re not managing at home and then you get the indication that they’re going to either need some other care home or you know they’d need to be put into a nursing home or something. (FY doctor 2)
		Quote 7. But I think the ones that are going to stay longer are the patients with a lot of comorbidities but also already have carers at home. (Registrar 1)
		Quote 8. A lot of the long stay patients would be the ones that have changed their discharge destination. (Nurse 7)
Therapy assessment	Functional and mobility assessment	Quote 9. I also think the therapy assessment as well, because when you go and see them and they’re sat in a bed, and you think actually they look great, and then physio gets them up and says they can’t stand, and suddenly that really, really sort of re-organises your thinking doesn’t it? (Registrar 3)
		Quote 10. Well I think they’re all at risk of functional decline. I think all of them, so which is why it’s so important that they all see physio. (Registrar 1)
	Patient’s engagement with therapy	Quote 11. I think you know you can in time you learn to identify those patients who present, because it is the ones that don’t really engage much. (Occupational therapist 2)
		Quote 12. If somebody’s older, with more comorbidities, and really motivated to get out of hospital, they do sometimes will do that, so I think there is a personality aspect, which we can sometimes pick up on. (Registrar 2)
		Quote 13. Sometimes patients with cognitive problems or dementia or whatever, if they have a decline in mobility then our patient can sometimes limit their rehab potential, if they have short-term memory problems or there’s no carryover between sessions and things like that, it can be quite difficult. (Physiotherapy 2)
Difficulties and challenges	Delirium	Quote 14. On acute admissions it is sometimes very difficult because there may be a delirium which confuses the issue (Consultant 2)
	Predicting risk of functional decline	Quote 15. What is harder to understand, is how much patients will decline functionally during their admission, and we can certainly take a best guess, but sometimes we are surprised that our best guess is not right.(Consultant 1)
	Communication of risk assessments	Quote 16. Routine measurements for example observations, MUST score, pressure areas, weights are not routinely communicated unless it is a major problem. Failing that when we do our ward rounds we make it a point to look at the bedside observation chart to get the information we need. (Consultant 2)
		Quote 17. It’s a very unfriendly document. There’s lots of writing, and it’s more of a tick-box exercise for them, and I feel bad for them that they have to complete it to be honest. (Registrar 2)
		Quote 18. Yeah, there is an awful lot of repeat isn’t there? But yeah I think generally we go on the medical clerking, I don’t think we generally look at them. (Registrar 3)
	Delayed clinical judgment	Quote 19. I guess in the people where it’s marginal and you then have a delay in making that assessment, if there was something immediately done on admission that identified that person, it would speed up the process and reduce their admission, (FY doctor 1)
		Quote 20. The person initially clerking the patient, could make a better assessment or a better move to getting collateral history I think that would save a lot of time further down the line. Failing that I think we are always approaching other members of the team to obtain collateral history from whatever other sources we can, so quite possible, but having it at the onset can save time later on. (Consultant 2)

#### Theme 1: “Clinical judgment”

Staff relied on their “clinical judgment” to interpret patient’s needs and to recognise those who might be at risk of poor healthcare outcomes. A number of factors influenced clinical judgment including: *reasons for admission, familiarity with patients, patient’s general condition, visible frailty, and patient’s ability to manage at home*. One consultant reported that severity of acute illness and high number of comorbidities as well as poor response to treatments enabled him to assess the likelihood of deterioration. Another consultant reported that familiarity with patients (see Quote 2, Table 1) assisted his judgment. Nursing staff stated that they recognised high-risk patients from their general condition. Visible frailty was reported to influence registrars’ clinical judgment (see Quote 4, Table 1). Similarly, dieticians described that they encouraged nursing staff to use their clinical judgment to recognise frail patients.

Consultants and therapists reported that patients’ inability to manage at home despite a care package was an important factor in judgment that these patients might need higher levels of care such as placement in a nursing home. Nursing staff and FY doctors stressed that the need to change discharge destination was the main predictor of prolonged length of stay (Quotes 6 and 8, Table 1).

#### Theme 2: “Therapy assessment”

Registrars reported that therapists’ assessment of patients played a crucial part in identifying older people at risk of functional decline or longer hospital stay (Quotes 9 and 10, Table 1). Therapy staff and registrars considered patients who lack motivation and fail to engage with therapy to be at higher risk of poor outcomes. Therapists recognised that disengagement could be sometimes due to inability to understand and follow instructions as a result of cognitive impairment such as dementia (Quote 13, Table 1).

#### Theme 3: “Difficulties and challenges”

Participants reported a number of challenges that could hinder their ability to identify high-risk patients including: *delirium, predicting risk of falls, delayed clinical judgment, and lack of communication.* One consultant reported that the presence of delirium made judgment of a patient’s true risk status more difficult (Quote 14, Table 1). Registrars and therapists reported that all patients were at risk of functional decline and deconditioning, hence the importance of therapy assessment and treatment. However, consultants believed that it was hard to predict how much patients will decline functionally during their admission and that they usually take a ‘best guess’ which can be wrong sometimes (Quote 15, Table 1).

Communication of the routine risk assessments between staff was a challenge. These assessments were not communicated to doctors unless there was a need for medical advice. Registrars reported that nursing notes were unfriendly documents with much repetition. Importantly, FY doctors and consultants reported needing several days to make a clinical judgment of patients’ needs which could potentially delay interventions (Quotes 19 and 20, Table 1). FY doctors and registrars thought the adoption of a simple clinical tool to highlight those high-risk patients earlier in the admission could be helpful.

### Quantitative results

A random sample of 60 patients’ (35 female) clinical records across the five study wards were reviewed. Patients’ demographic variables are detailed in Table 2. This group of patients (mean age 86.7 years) had complex needs and multiple risk factors: 42 (70%) had more than five active comorbidities at time of admission and 51 (85%) were taking more than five medications; 50 (83%) had both high comorbidities and polypharmacy. Forty-six (77%) of patients had dementia and 35 (58%) had do not attempt resuscitation (DNAR) forms completed. Twenty-four (40%) of patients were discharged to a new destination; more than half of these went to nursing homes and six died.

**Table 2:** Characteristics of participants

Patients’ characteristics [number (%)]	Patients
	N = 60
Age (years) (Mean ± SD)	86.7 ± 5.3
Sex	
Male	25 (42%)
Female	35 (58%)
Usual residency	
Lives home alone	23 (38%)
Lives home with friends or relatives	17 (28%)
Sheltered accommodation	3 (5%)
Residential/rest home	9 (15%)
Nursing home	8 (13%)
No of comorbidities	
Median	6
Ranges	4–14
Number of medications	
Median	9
Ranges	3–16
Patients with dementia	46 (77%)
DNR report	35 (58%)
Length of stay (days)	
Median	24
Ranges	(4–76)
Discharge destination	
Usual residence	36 (60%)
New residence	24 (40%)
Rehabilitation units	1 (4%)
Nursing home	13 (54%)
Other hospitals	4 (17%)
Patient died	6 (25%)

*N* number, *%* percentage, *SD* standard variation, *IQR* interquartile range, *DNR* do not attempt resuscitate report

A number of routine risk assessments and relevant care plans were completed by nursing staff on admission (see Table 3). These included assessment of nutrition (completed for 85%), falls risk (95%), mobility (85%), and pressure ulcer risk (88%). Eight (16%) patients were found to be at high-risk of malnutrition but in fact 11 (18%) were referred to the dietetic team and 19 (32%) patients were prescribed oral nutritional supplements. Forty-eight (80%) of patients were seen by the therapy team early on admission. Only two patients were recorded to be frail. No specific tool was used for the assessment of frailty.

**Table 3:** Routine risk assessments performed on admission to hospital wards

Risk assessments	Number of patients assessed (%)	Score n (%)	Assessment within 3 days of admission n (%)
Assessment of nutrition	51 (85%)	Higher risk = 8 (16%)	36 (71%)
		Lower risk = 43 (84%)	
Assessment of falls risk	57 (95%)	High risk = 36 (60%)	51 (90%)
Handling and mobility assessment	51 (85%)	Independent = 12 (24%)	47 (92%)
		Requires assistant = 28 (55%)	
		Dependent = 11 (22%)	
Assessment of pressure ulcers risk	53 (88%)	At risk = 34 (64%)	50 (94%)
		Moderate risk = 8 (15%)	
		High risk = 8 (15%)	
		Very high risk = 3 (6%)	

No significant association was found between risk of malnutrition, falls and pressure ulcers and discharge destination. Similarly risk of malnutrition, pressure ulcers and handling and mobility risk assessments were not associated with length of stay. However, there was a significant association between risk of falls and length of stay in this small sample.

## Discussion

This study described how older inpatients at higher-risk of poor healthcare outcomes such as longer length of stay in hospital, reduced physical function, and discharge to care home were identified in routine hospital practice. Staff relied on their “clinical judgment” and “therapy assessment” to recognise high-risk patients. The multi-disciplinary setting (medical, therapy, and nursing staff) appeared to facilitate clinical judgement. However, a number of challenges in making this clinical judgment were reported leading to possible delays. Few risk assessment measures completed routinely by nursing staff on admission to the wards. Yet, the purpose of these assessments was to identify the risk of a single adverse outcome and they were not used collectively to highlight patients at risk of poor healthcare outcomes. Patients’ clinical records lacked documentation of staff clinical judgment.

Clinical judgment can be defined as “an interpretation or conclusion about a patient’s needs, concerns, or health problems, and/or the decision to take action (or not), use or modify standard approaches, or improvise new ones as deemed appropriate by the patient’s response” [11]. It is complex and requires a flexible ability to recognize prominent aspects of an undefined clinical situation, interpret their meaning, and respond appropriately. It relates to the experience of individual clinicians. Senior clinicians often possess experience and knowledge to inform their decisions. Junior clinicians might be less confident in their clinical judgment and tend to follow protocols to eliminate variability in patient care [12]. Similarly in our study, there was a discrepancy in practice between consultants and junior doctors which in turn could have impacted on their clinical judgement.

There are concerns about the accuracy of clinical judgment on the functional status of older patients in hospitals, and to predict length of stay and survival from critical illness and mortality [13, 14]. Some staff interviewed in this study questioned the accuracy of their clinical judgment sometimes suggesting that a simple measure could be highly relevant in daily practice. This measure should be easy to use in clinical settings, quick, cheap and reliable. A number of assessment instruments have been developed to predict risk of adverse outcomes among hospitalised older inpatients [15–18]. However, they have not been proven to be accurate and reliable [19].

Gait speed has been recommended as the most suitable assessment tool to be implemented in standard clinical evaluation of community dwelling older people [20]. However in hospital where many patients are acutely ill and unable to walk, grip strength measurement could be more suitable [21]. Low grip strength is associated with poor current and future health including increased falls [22], morbidity [23], and death [24]. Further research is needed to evaluate the feasibility of its implementation in routine clinical practice [25].

## Limitations

This study was conducted in one hospital in England and the current practice identified in this study may not be necessarily generalised to other hospitals and departments. Therefore, it is important to replicate this work to compare variation in clinical practices. Second, the study reported only what was documented in clinical records and in staff interviews. Discussions between clinical staff during the ward rounds and multi-disciplinary team meetings were not captured. However, we believe that the interviews and focus groups with multiple healthcare professionals addressed this concern.

## Conclusions

Staff relied on their clinical judgment to identify high risk patients. Making such a judgment was often difficult and could occur several days after admission potentially delaying specific interventions. A number of risk assessments were carried out routinely but used mainly to identify single healthcare needs rather than holistically to identify individuals at risk of poor healthcare outcomes to the multi-disciplinary team. We believe that early identification of those patients using a valid measure alongside staff clinical judgment could be highly relevant. Future research will assess the feasibility and acceptability of using grip strength measurement as a tool to identify older patients at risk of poor healthcare outcomes.

## Additional file

**Additional file 1.** Semi-structured guide for interviews/focus groups.

## Abbreviations

FY: foundation year
DNAR: do not attempt resuscitation
