# Supplementary material for: Can routine clinical data identify older patients at risk of poor healthcare outcomes on admission to hospital?
Source: BMC Res Notes. 2017 Aug 10;10:384. doi: 10.1186/s13104-017-2705-7 (PMC5553791; doi:10.1186/s13104-017-2705-7)
Supplement: Supplementary file 1 — Additional file 1. Semi-structured guide for interviews/focus groups. [file 13104_2017_2705_MOESM1_ESM.docx]

**Can routine clinical data identify older patients at risk of poor healthcare outcomes on admission to hospital?**

**Semi-structure interview/focus group schedules**

**Interview/** **focus group schedule for nursing and medical staff**

1-Can you tell me about your role in relation to the care of older people? How many years of experience do you have in this field?

2- Could you talk me through what happens when a new patient is admitted to the ward? What are the main routine assessment measures in place?

3- Can you normally identify patients at risk of long length of stay (LOS) or discharge to care home early in their admission to hospital?

4- Could you tell me when and why a patient would be referred to a dietician? or for physiotherapy?

5- What would you think of a simple measurement that could identify those patients at risk of poor health outcomes at admission to the ward? Would you like to use it? Why?

6. Do you know about grip strength measurement? what do you think of using GS in routine practice?

7. How do you think GS measurement could be integrated routinely into the admission procedures? What are the different (negative and positive) factors that could impact its implementation?

**Interview schedule/** **focus group for dietetic team**

1. Can you tell me about your role in relation to the care of older people? How long have you been doing this?

2. What is the normal dietetic care provided to patients? Are there any challenges in the dietetic care provided to older patients?

3. Is there any screening tool used routinely to assess the nutrition status of inpatients?

4. When are you asked to review a patient in MOP? Who makes the referral to you?

5. Can you talk me through what kind of interventions (pharmacological and non-pharmacological) or care plans are available to manage patients at risk of malnutrition?

6. What is your aim when you start a care plan or an intervention? How do you measure the outcomes?

**Interview/** **focus group schedule for therapy team**

1. Can you tell me about your role in relation to the care of older people? How long have you been doing this?

2. Could you tell me what is the usual mobility care provided to patients in MOP’ wards? Are there any challenges in providing this care to patients?

3. When are you asked to review a patient in MOP? Who makes the referral to you?

4. Can you tell me how you prioritise your visits? How do you assess patient’s mobility levels?

5. Can you talk me through what kind of therapies are available to manage the patients?

6. What is your aim when you start a care plan, an intervention, or a programme for a patient? How do you measure the outcomes?

7. Progressive resistance training has been suggested as a therapeutic treatment for low grip strength. So I would like to seek your views about the feasibility of using progressive resistance training among older inpatients?
